# Torso hemorrhage: noncompressible? never say never

**DOI:** 10.1186/s40001-024-01760-4

**Published:** 2024-03-06

**Authors:** Lian-Yang Zhang, Hua-Yu Zhang

**Affiliations:** Department of Trauma Surgery, War Trauma Medical Center, State Key Laboratory of Trauma, Burn and Combined Injury, Daping Hospital, Army Medical University, Chongqing, 400042 China

**Keywords:** Noncompressible torso hemorrhage, External hemostasis, Trauma, Prehospital emergency

## Abstract

Since limb bleeding has been well managed by extremity tourniquets, the management of exsanguinating torso hemorrhage (TH) has become a hot issue both in military and civilian medicine. Conventional hemostatic techniques are ineffective for managing traumatic bleeding of organs and vessels within the torso due to the anatomical features. The designation of noncompressible torso hemorrhage (NCTH) marks a significant step in investigating the injury mechanisms and developing effective methods for bleeding control. Special tourniquets such as abdominal aortic and junctional tourniquet and SAM junctional tourniquet designed for NCTH have been approved by FDA for clinical use. Combat ready clamp and junctional emergency treatment tool also exhibit potential for external NCTH control. In addition, resuscitative endovascular balloon occlusion of the aorta (REBOA) further provides an endovascular solution to alleviate the challenges of NCTH treatment. Notably, NCTH cognitive surveys have revealed that medical staff have deficiencies in understanding relevant concepts and treatment abilities. The stereotypical interpretation of NCTH naming, particularly the term noncompressible, is the root cause of this issue. This review discusses the dynamic relationship between TH and NCTH by tracing the development of external NCTH control techniques. The authors propose to further subdivide the existing NCTH into compressible torso hemorrhage and NCTH’ (noncompressible but REBOA controllable) based on whether hemostasis is available via external compression. Finally, due to the irreplaceability of special tourniquets during the prehospital stage, the authors emphasize the importance of a package program to improve the efficacy and safety of external NCTH control. This program includes the promotion of tourniquet redesign and hemostatic strategies, personnel reeducation, and complications prevention.

## Background

Hemorrhage is the leading cause of potentially preventable death (PPD) during wartime. The efficacy of torso hemorrhage (TH) control is increasingly becoming the key to reduce prehospital mortality in the context of the widespread use of limb tourniquets. The popularization of existing treatment methods is conductive to further upgrade hemostatic strategies in the “bedside-bench-bedside” cycle. With the advent and optimization of tourniquets, distinctions between noncompressible torso hemorrhage (NCTH) and TH have inevitable arisen. Due to the lack of guidelines and consensus regarding to TH or NCTH, it is of importance and necessity to summarize the historical evolution of NCTH definition and corresponding external hemostatic strategies. Hence, the clarification of NCTH and TH is essential for reducing the conceptual misunderstandings as well as orienting continuing training programme.

## War and torso hemorrhage: the Achilles’ heel of combat casualty care

The chessboard resuming of war fatalities is always tinged with regret and helplessness. However, as a decisive cornerstone of improving the casualty care system, the results provide insights into the hotspots of war injury prevention, comprehensive treatment and first-aid provider training. The military has always attached great importance to the construction of trauma database [1–3]. Based on data analysis represented by the Department of Defense Trauma Registry has profoundly influenced and changed the breadth and depth of military medical research and practice over the past decades [4–7].

The ancient European proverb “war, the feast of death” reflects the inherent cruelty of war. The continuous upgrade of weapons in modern war further aggravates the complexity of injuries and the difficulty of treatment and rehabilitation [8–10]. Although the U.S. military case-fatality rate in Afghanistan and Iraq was at a record low of 9.5 percent, the high prehospital to hospital death ratio of 2.7 to 1 still exposed the shortcomings of the tactical treatment phase [11]. The mortality rate of casualties who died before reaching a medical treatment facility (MTF) was reported to be as high as 87.1–100.0% [12–16]. 91.0% of PPD was due to hemorrhage [12, 17, 18], 33.2% of which could be categorized as tourniquetable [19]. Noncompressible cases accounted for 70.3–80% of hemorrhagic deaths [19, 20], with the thoracic and abdominopelvic cavities being the most vulnerable areas [12, 14].

Noncompressible hemorrhage was first proposed by Klemcke in 2006 [20], which is mainly used to describe bleeding from thoracoabdominal region that cannot be controlled via manual compression. Subsequently, hemorrhage was further classified according to bleeding sites and hemostatic methods: (A) torso: NCTH; (B) extremities and junctional regions (axilla, groin, etc.): conventional tourniquets and other compression measures controllable hemorrhage. Finally, Morrison and Rasmussen systematically summarized the definition, anatomical features, diagnosis and treatment principles of NCTH [21, 22].

**Fig. 1:**
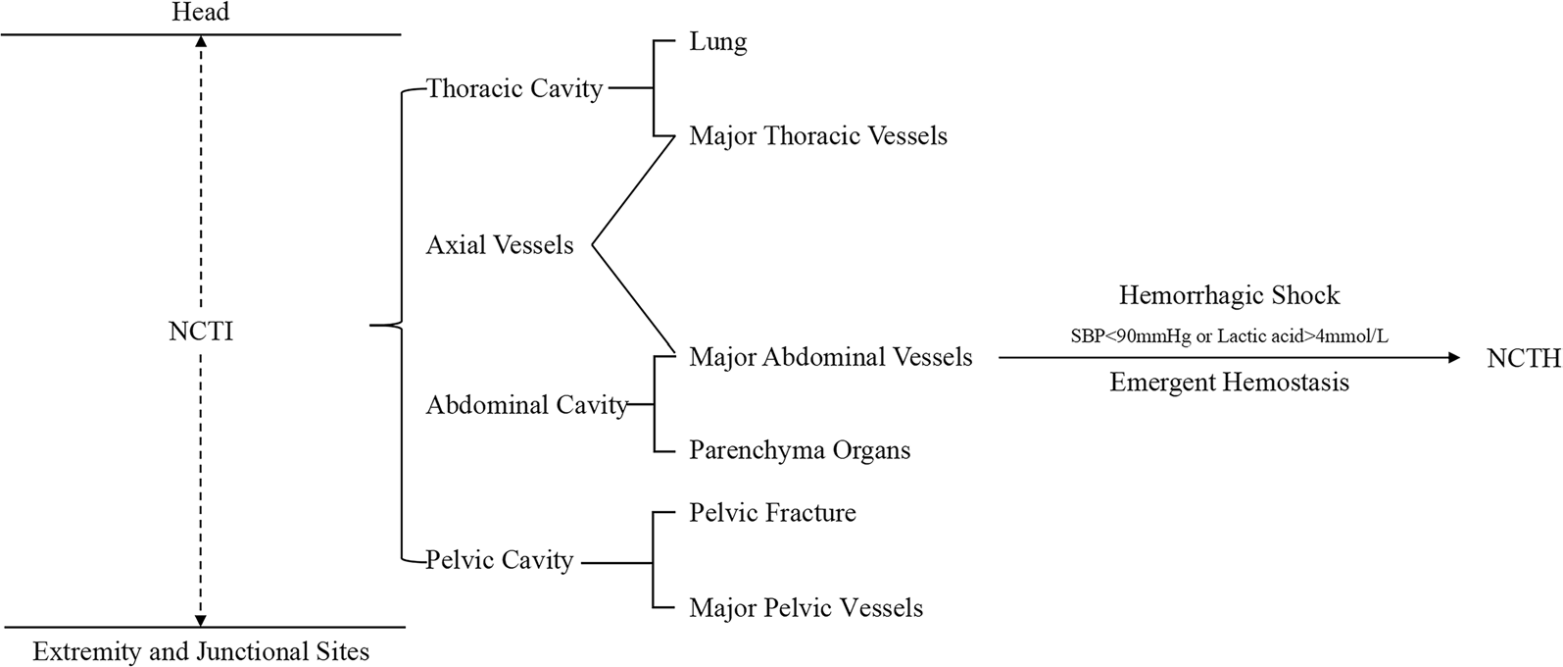
The sketch map of the relationship between NCTI and NCTH. NCTI: noncompressible torso injury, NCTH: noncompressible torso hemorrhage, SBP: systolic blood pressure

Noncompressible torso injury (NCTI) is defined as trauma to organs or vessels within the anatomical range of torso. The resultant hemorrhagic shock and impaired physiological reserve leads to NCTH, and immediate hemostatic treatment are urgently needed (Fig. 1).

According to a 10-year observational study, a total of 296 British military personnel were diagnosed with NCTH, prehospital deaths were 7.1 times higher than deaths occurred in MTF with an overall mortality rate of 85.5%, which was 2.1 times higher than that of non-NCTH casualties [23, 24]. The leading cause of NCTH death was explosive (69.6%) artery injuries (60.1%). Nearly 20.0% of NCTH deaths resulted from irretrievable arterial (26.8%) and pulmonary (56.5%) exsanguination, even though definitive hemostatic treatment was received.

Personal protective equipment (PPE) such as helmets and body armor provide necessary protection for the combatants [25–28]. However, limbs and torso are still the most vulnerable sites of dismounted complex blast injuries on the battlefield where improvised explosive devices are increasingly used [29, 30]. This phenomenon reflects that the process of PPE from “basic protection” to “ideal protection” still contains many technical problems to be solved [26]. To fully achieve the flexibility of individual tactics, warfighters rarely use PPE for limb protection. And the resulting destructive limb fragmentation and exsanguination are still common [31–33]. Encouragingly, owing to the efforts of Tactical Combat Casualty Care regarding to the tireless promotion of standardized battlefield rescue [34, 35], limb tourniquets have been extensively deployed in frontline troops [36–38]. The death rate of extremity hemorrhage dropped by an astonishing 85.0% [39]. Accordingly, how to effectively reduce the death rate of TH, particularly filling the gap between NCTH control and the awkward situation of high PPD in tactical stage, has become a thorny task of war trauma research. Even more, NCTH control is dubbed “the ultimate challenge” in the field of tactical rescue [40].

## Civilians and torso hemorrhage: the sword of Damocles in peacetime

Trauma is not an exclusive adjunct to war, which also poses great challenges to human health in peacetime. According to Global Burden of Disease Study 2017, more than 500 million people worldwide suffered trauma resulting in more than 4 million deaths [41]. Besides, Corso and colleagues revealed that more than 50 million American citizens received medical services for traumatic events, and the cost of treatment and labor loss was staggering $406 billion [42].

America’s population grew by 9.7% between 2000 and 2010, but the increase in trauma deaths climbed to 22.8%. Trauma has become a key cause of death in the elderly population since mortality from cancer and cardiovascular disease has declined significantly over the same period [43]. As the aging population continues to increase, it is reasonable to believe that more elderly people will suffer from trauma with a decreasing life expectancy thereby [44]. Unfortunately, compared to the huge amount of budget spent on oncology research, less than a tenth of that was invested into the field of trauma care study [43].

In peacetime, the prehospital trauma mortality ranged from 36.3% to 71.0% [45–48], and the prehospital PPD incidence was as high as 92.4% [48]. Unlike war trauma, motor vehicle collision is the main mechanism of injury in peacetime [49, 50]. However, the so-called axial region of human body is still the most vulnerable site [51, 52], and hemorrhage and shock account for approximately 37.0–55.1% PPD cases [53–55]. In addition, Branco reported that the incidence of abdominal aortic injury increased more than twice in 2014 compared with 13 years ago (2014: 66.0% vs 2002: 30.4%, *p* < 0.001) [56]. The data not only confirm the researches on the fatal spots of malignant injuries [57, 58], but also re-emphasize the great potential of effective control of TH in alleviating PPD. Undoubtedly, prehospital stage is still the “critical window period” for hemorrhage control and PPD inhibition [59, 60].

According to an epidemiologic study that covered nearly all level 1 trauma centers in the United States [61], the incidence of NCTH was 8.2% among 241,904 NCTI patients. The mortality rates of NCTI and NCTH were 6.8% and 44.6%, respectively, conforming a higher degree of NCTH severity than NCTI. Penetrating wounds caused by gunshot were the primary mechanism of NCTH, and lung (68.1%), axial vessels (67.8%), parenchyma viscera (35.3%) and pelvis (9.2%) were the top 4 susceptible sites. When combined with risk factors such as advanced age, disturbance of consciousness, higher injury severity score and penetrating injury, NCTI patients were not only more likely to progress to NCTH, but also correspondingly increased the possibility of blood transfusions and mortality risk.

Another multicenter study reported a 26.2% mortality rate of NCTH, and exsanguination as the primary cause of death was also revealed [62]. Meanwhile, descending aorta (45.3%), renal artery (10.7%) and internal iliac artery (43.7%) were the most easily involved vessels in thoracic, abdominal and pelvic cavity, respectively. Subsequently, Italian researchers highlighted the urgent importance of early hemostatic intervention via revealing that the majority of NCTH mortality occurred in the early stage of injuries [63].

## Torso hemorrhage and cognition of medical staff: time to break the information cocoons

A series of knowledge, attitude and practice (KAP) survey implied that better commands of key knowledge such as definition and diagnosis may produce more positive attitude and adequate management [64–66]. However, the amount of NCTH-knowledge related studies is still insufficient [67–69], which not only increases the difficulty of obtaining the overall cognitive level, but also reflects the current situation that NCTH-related concepts have a relatively low clinical acceptance with fewer practitioners. Therefore, how to overcome the shortage of NCTH popularization should be deemed as an important component to improve the treatment efficacy systematically.

According to an online KAP survey among Chinese medical staff [67], the vast majority of respondents met the positive evaluation criteria correlated with the holistic NCTH knowledge (94.3%) and attitude (99.7%). However, there were still many cognitive and operational deficiencies that did not match the positive attitude through the analysis of each topic. On one hand, an obvious contradiction existed between respondents’ misunderstanding of NCTH definition and the existing tourniquets. 87.1% of the questionnaires indicated that NCTH was a type unique hemorrhage, which could not be controlled by any tourniquets. Meanwhile, more than half of respondents also believed that external NCTH control had become a reality. The one-sided understanding of “noncompressible” may explain this phenomenon. On the other hand, medical staff demonstrated inadequate knowledge of NCTI anatomy and NCTH diagnostic criteria. 40.2% of respondents did not consider chest trauma as an essential component of NCTI, whereas the proportion of people who included injuries of hollow organs and small vessels as NCTI was 79.1% and 63.1%, respectively. And only half of the respondents acknowledged the value of lactic acid content in NCTH diagnosis. Accurate understanding of the diagnostic criteria is the key to reduce missed diagnosis and misdiagnosis. Due to the lack of unified cognition of NCTH, the reported annual NCTH treatment rate of 48.0% should be viewed more cautiously, which addressed the urgency of NCTH epidemiological investigation in China. The authors also pointed out that relevant training and literature reading were essential in the improvement of clinical skills and attitudes. However, the significant shortage of specific training was warranted of concerns for better clinical outcomes.

The same research team further explored doctors’ cognitive level of NCTH, as well as attitudes and preferences towards continuing training after completing a self-administered study program [68]. Notably, the correct answer rate of NCTH definition (20.0%), as well as the involved organs (33.9%) and vessels (37.2%) was still similar to the previous study after completing the handout reading. Given that most respondents more inclined to improve their NCTH control skills, hence offline hand-on training (67.8%) were more preferred, whereas online self-learning (1.1%) was the least popular training mode. The results suggested that theoretical self-study alone was not that effective in reversing the wrong cognition of NCTH. Therefore, Practical training and case studies involving thoracic and abdominopelvic hemostasis may be more beneficial to break the current NCTH information cocoons. Similarly, in another survey closely related to NCTH treatment [69], respondents also showed their willingness to further improve the hemostasis ability through training, and identified the main barriers to participating in continuing training such as fees and time costs. Consequently, how to stimulate the participation enthusiasm of trainees puts forward higher requirements for training organizers.

The above studies imply the urgency of continuing NCTH training and the complexity in optimizing training pattern and contents. It is precisely because of the tough process from germination to maturity, more attention should be paid to the value of goal-directed education [70, 71]. Giving full play to the guiding role of existing data, rather than simply appealing to improving clinical importance may exert more practical outcome.

## Torso hemorrhage and its noncompressibility: nothing is impossible

The reasons why torso is a vulnerable region for PPD are as follows: (A) Torso is the central site of the human body with a relatively large surface area, which is prone to be the target of various injury factors. (B) The lack of frontal bone protection makes intraperitoneal tissues and organs are more susceptible to trauma. (C) The dilemma of hemostasis—the low availability of tourniquets and the high level of expertise in intracelial hemostasis—significantly raises the threshold for on-site and evacuation rescue. Therefore, the research focus of TH naturally divides into two aspects: enhancement of protection and innovation of hemostatic measures. The latter is more likely to play an active role in both wartime and peacetime trauma treatment.

**Fig. 2:**
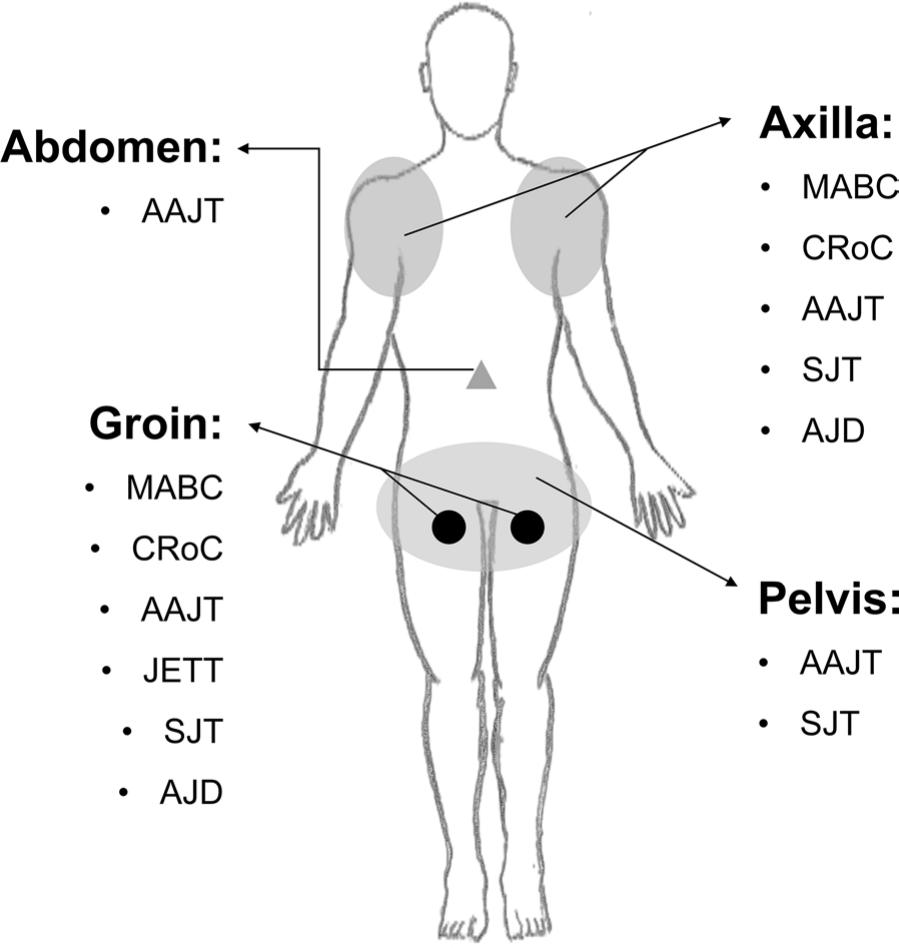
The application scope of hemostatic tourniquets. MABC: modified adjustable bar clamp; CRoC: combat ready clamp; AAJT: abdominal aortic and junctional tourniquet; JETT: junctional emergency treatment tool; SJT: SAM junctional tourniquet; AJD: Adonis junctional device

At present, there are at least 6 tourniquets that can be applied for torso and its junctional regions (Fig. 2) [72–75]. Although the shapes are different and some tourniquets are originally designed for junctional hemostasis, applying additional force to block blood flow from the bleeding site or its upper stream is the shared philosophy of hemostasis. In addition, continuing researches further expand the application scenarios and scopes of tourniquets [76–78]. Therefore, the potential benefits of junctional tools in TH control deserve more attentions. It is even possible to completely break up the boundary between TH and junctional hemostasis in the future. Based on this, the authors believe that it is necessary to summarize the key features of existing tourniquets, so as to provide necessary information for the iteration and innovation of novel devices.

Generally speaking, current tourniquets can be regarded as the derivative of the Abdominal Tourniquet (AT) invented by Lister in the eighteenth century [75]. The prototype of AT is made of metal, which is shaped like a capital letter “D”. Although AT did not play the expected role of controlling hip bleeding in clinical practice, it made a pioneering attempt to establish external control strategies for TH.

The boom in industrial design and materials science made up for Lister’s regret. A novel tourniquet, called Modified Adjustable Bar Clamp (MABC), is designed to control the increasing junctional hemorrhage in the battlefield [75]. The double-ended pressurized elements facilitate flexible placement of MABC according to the anatomical features of targeted sites, thus improving the portability and operability of the device. However, due to the lack of evidence-based studies, the actual efficiency and physiological parameters are still unavailable at present.

Combat Ready Clamp (CRoC. https://combatmedical.com) is a tourniquet that closely resembles AT in both shape and operation mode. Several studies have confirmed the efficacy and stability of CRoC in inguinal and axillary hemostasis [79–82]. When placed onto the lower abdomen, the required pressure (625 ± 8 mmHg) is greater than that for the groin (524 ± 12 mmHg) due to the obstruction of CRoC compression conduction to deep vessels by abdominal wall tissues [77, 78]. Although the pressure required by CRoC to block the flow of aortic bifurcation is much higher than the default pressure limit (300 mmHg) of Abdominal Aortic and Junctional Tourniquet (AAJT. https://compressionworks.com) [72], the successful umbilical application of CRoC on cadavers is still a model to broaden the application boundary of junctional tourniquets [78]. Human studies have revealed that short-term application of CRoC did not cause significant complications [83]. However, large animals may suffer from long term dysfunction caused by obvious ischemia and reperfusion injury (IRI) when blood occlusion had to be prolonged [77].

AAJT is the only tourniquet appropriate for both torso and junctional bleeding control in the last decade. The user-friendly design including the wedged airbag, visualized and overload-protecting valve, as well as ratchet or twist fastening element enhances the efficacy and safety of AAJT. Although pain during hemostasis is the biggest obstacle to the successful completion of the volunteer studies [81, 83], the clinical case of blood control and the positive feedback from the survivor suggest the applicability and indispensability of AAJT toward unconventional rescue [84].

In general, AAJT can reduce the amount of fluid resuscitation [85]. But the unnecessary compression on tissues beneath the airbag and intestine dislocation are somewhat inevitable [85]. The iatrogenic elevated intra-abdominal pressure may ultimately progress to deaths of spontaneous breathing animals from cardiopulmonary failure after the deflation of balloon [86]. Laboratory data have shown that animals were survivable when AAJT application time extended to 4 h [85]. However, necrosis of the liver and intestine suggests that prolonged prehospital phase is a secondary strike to the patients, which once again highlights the significance of “golden hour” in trauma treatment [87]. Compared with AAJT, Resuscitative Endovascular Balloon Occlusion of the Aorta (REBOA) has a higher popularity attributed to its potential in alleviating IRI [88, 89]. Bridging the application of AAJT to REBOA may strive a safer transition from prehospital to hospital hemostasis and better survival benefits [90, 91].

Junctional Emergency Treatment Tool (JETT. https://narescue.com) consists of a fastening belt and two identical pressurized screws. Several studies have shown that JETT was less efficient than other tourniquets in unilateral hemostasis [83, 92, 93]. However, when focused on bilateral hemostasis, the advantage of JETT was demonstrated [94]. Radiographic evidence suggests that JETT can reduce the volume of fractured pelvis (APC III) via annular contraction [95], which further expands its use in pelvic fixation.

SAM Junctional Tourniquet (SJT. https://sammedical.com) is close to JETT in overall appearance. Unlike the single large airbag of AAJT, SJT has a much smaller balloon in size, which significantly reduces the unnecessary compression of the surrounding tissues. When necessary, SJT can be used as a pelvic fixator or axillary hemostat with a shoulder accessory. Because of its efficacy and relatively mild pain [73, 81], SJT is more favored among tourniquets [73, 83, 96]. Regrettably, due to the lack of clinical and laboratory data, the positive role of SJT in air transport has been solely clarified. More details correlated with physiological changes and long-term adverse effects remain to be determined [97].

Adonis Junctional Device (AJD) is a newly reported tourniquet [74]. This device, named after Adonis—the Greek mythological figure who died of groin trauma, not only vividly embodies the purpose of AJD, but also reflects the humanistic spirit of designers. Even though AJD has similar physiological effects with other tourniquets plus no obvious IRI has been detected via pathological examination, attentions should be paid to the long-term risk of limb dysfunction according to the phenomenon that the pressure of the affected side remains lower than that of the non-affected side.

## The intrinsic philosophy of torso hemorrhage: who am I and where am I going

When Lister puts forward the concept of torso compression, medical scientists represented by Pancoast also carried out a series of AT-related design and performance tests independently [98, 99]. Since then, a great deal of evidence suggests that TH has been changed from “whether it is compressible” to “what type of TH is compressible” and “how to better develop the potential of tourniquets” based on the fact such as the pioneering attempt of high-plane hemostasis of CRoC in the abdomen [77], the validity of JETT, SJT and AAJT in pelvic fractures [95], and the similarity of external compression and endovascular hemostasis [100].

Abandoning the old-school thinking that TH cannot be controlled via external compression and taking full advantage of tourniquets in emergent TH control is practical to improve the survival benefit and ultimately achieve the best practice in prehospital trauma care. However, the misunderstanding of NCTH’s definition [67, 68], especially the literal understanding of the word “noncompressible”, which not only reflects the necessity and urgency of NCTH-related training, but also indicates the relative mismatch between NCTH naming and current development of hemostatic technology.

**Fig. 3:**
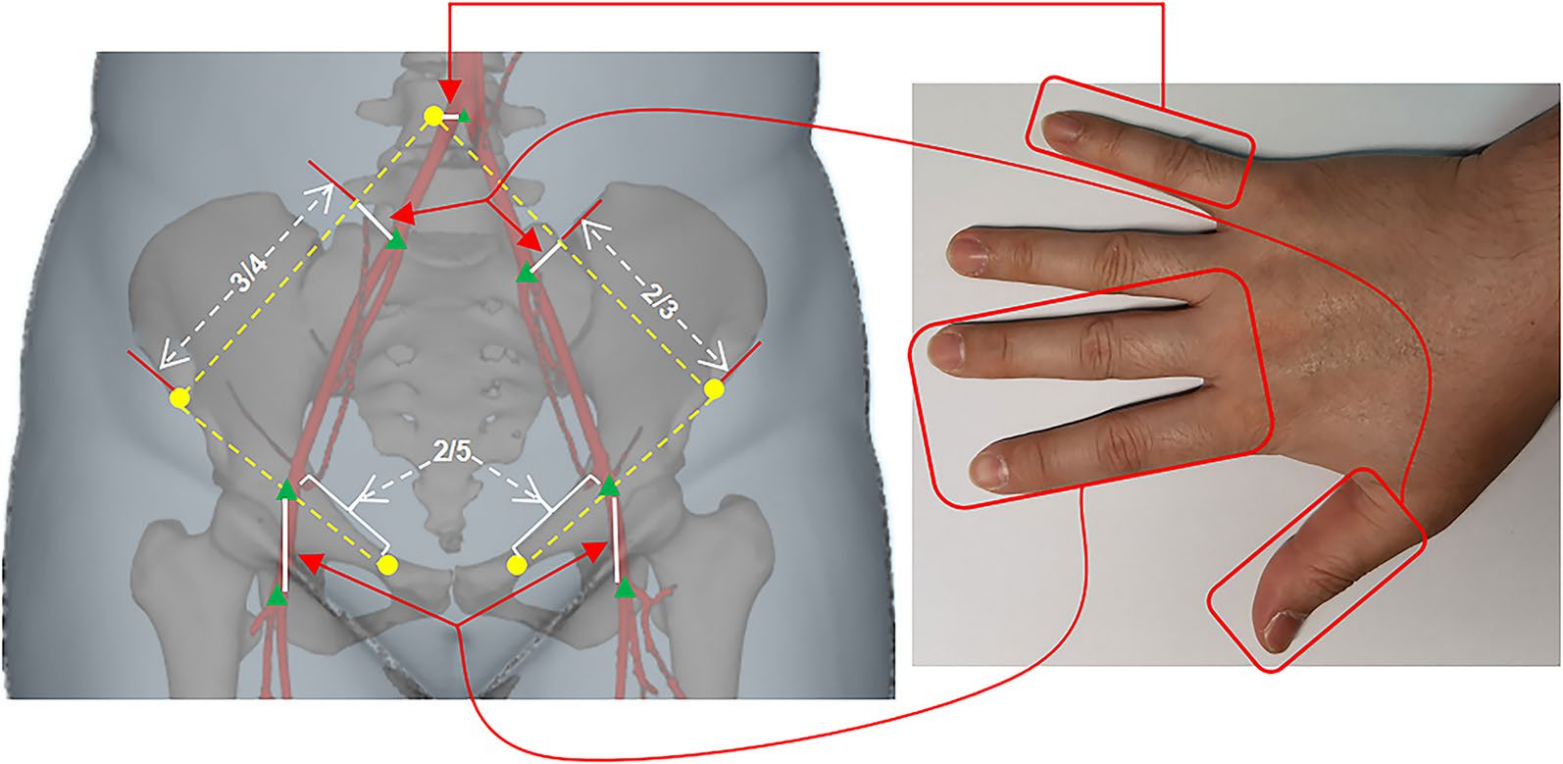
The surface localization strategy of abdominopelvic arteries. Yellow dots, respectively, represent landmarks of the umbilicus, the anterior superior iliac spine, and the pubic tubercle; green triangles, respectively, represent the aortic bifurcation and the termini of internal/external iliac artery and common femoral artery; female’s common femoral artery can be located 2 finger widths vertically below the medial 2/5 of the ipsilateral anterior superior iliac spine-pubic tubercle line

**Fig. 4:**
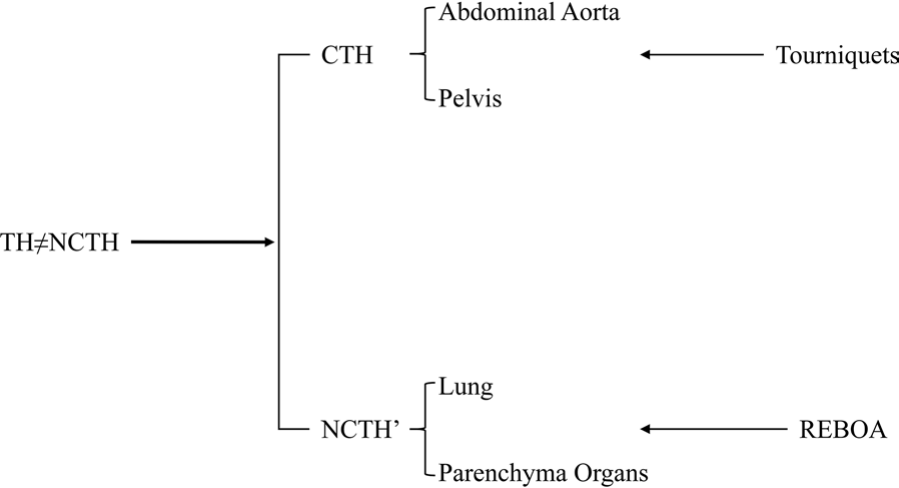
The definitional shift of TH and NCTH. TH: torso hemorrhage; NCTH: noncompressible torso hemorrhage; CTH: compressible torso hemorrhage; NCTH’: remaining NCTH; REBOA: Resuscitative Endovascular Balloon Occlusion of the Aorta

REBOA successfully compensated the deficiency that tourniquets are invalid in pulmonary and parenchymal hemorrhage. With the updating of hemostatic strategies such as partial REBOA (partially deflating the balloon for proximal permissive hypotension and titrated distal organ perfusion) and intermittent REBOA (periodically deflating the balloon for IRI postconditioning) as well as the improvement of catheterization techniques [101–103], more secure REBOA application have been advanced to prehospital scenarios. Paradoxically, a multicenter study conducted in the UK indicated that the introduction of REBOA did not improve survival in patients with exsanguinating bleeding and may even had the opposite effect [104]. Hence improving the survival benefits of REBOA is also significantly affected to the capacity of emergency medicine. Meanwhile, the inherent ease-to-operate nature of tourniquets greatly reduces the requirements for specific medical background as well as the training difficulty. In addition, combining with the potential of precise hemostasis implied by the novel arterial surface localization strategy, tourniquets are still the backbone of prehospital emergent hemostasis (Fig. 3) [105]. Furthermore, the development of polymer materials, novel hemostatic powder and nano agent has also boosted the methodology and materialogy of NCTH control [106–108]. Therefore, TH is no longer equal to NCTH, and the updated NCTH definition should exclude the bleeding sites that have already controllable via tourniquets. In summary, TH can be further divided into compressible torso hemorrhage (CTH) and the remaining NCTH (NCTH’) according to the feasibility of tourniquets. In other words, *TH* = *CTH* + *NCTH’* (Fig. 4).

**Fig. 5:**
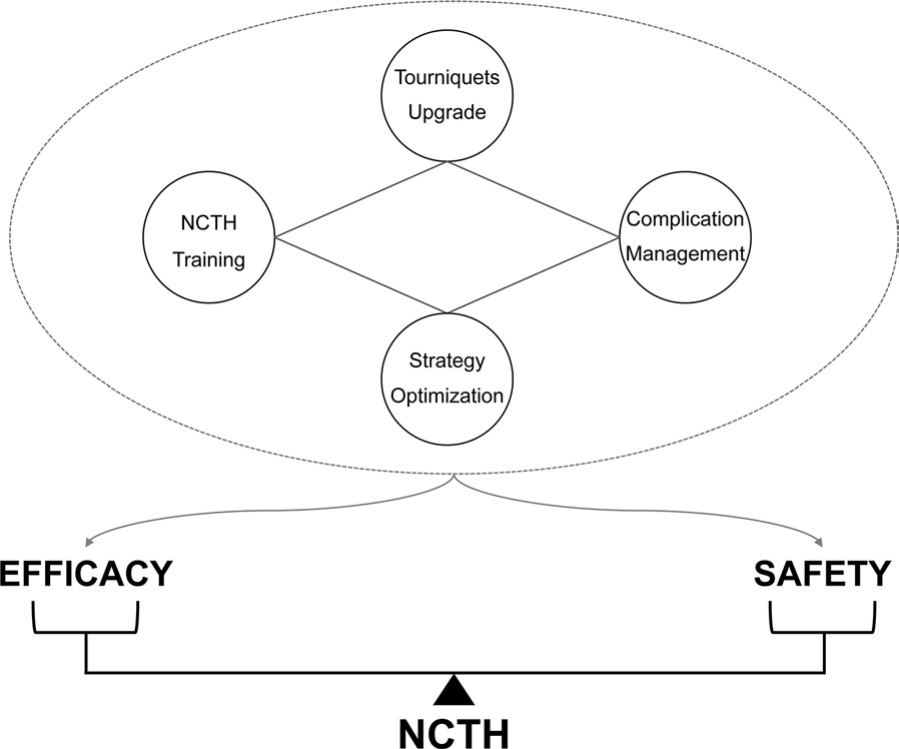
Prospects of ways to improve the efficacy and safety of external NCTH control. NCTH: noncompressible torso hemorrhage

How to better exert the efficacy of hemostasis and reduce the incidence and severity of complications should be the focus of future research on tourniquets redesign and strategy optimization (Fig. 5). Relying on a mature trauma training platform to popularize the basic concepts, academic progress and consensus of NCTH is helpful for increasing the awareness and acceptance of tourniquets among medical staff [109, 110]. And the on-hand training is the key to strengthen the clinical effectiveness. The elevation of the integration and refinement of tourniquets can reduce the application time and adverse events from the perspective of hardware, and thus improve rescue providers’ confidence. Besides, the progress of material science can produce positive significance of hemostatic stability and barrier-free imaging examination. IRI studies have confirmed the existence of the co-injury axis of intestine-lung and intestine-brain [111, 112], but more data are needed to verify the relationship of tourniquets in vivo working duration and the severity of distant organ lesions. Moreover, both partial and intermittent REBOA demonstrate the important role of permissive hypotension [113]. The combination of prehospital damage control resuscitation and external hemorrhage control techniques is promising in reducing mortality rates and improving the quality of patient recovery [113, 114]. Due to the lack of practical data on external NCTH plus permissive hypotension (similar to partial or intermittent REBOA), this statement aims to motivate further improvement of the overall efficacy and safety of external NCTH control.

## Conclusion

The ancient Chinese philosophy of “Yin Yang” profoundly reflects the complexity of eternal coexistence and mutual restriction of objects. The effective control of limb bleeding highlights the importance of prehospital TH control, which is a vivid embodiment of “Yin Yang”. Similarly, with the advent of various kinds of tourniquets, the uniform but diverse pattern of TH and NCTH also reflects the dialectical unification of “Yin Yang” as well as the relativity of “noncompressible” of TH.

## Data Availability

The corresponding author can be contacted upon reasonable request.

## Abbreviations

PPD: Potentially preventable death
TH: Torso hemorrhage
NCTH: Noncompressible torso hemorrhage
MTF: Medical treatment facility
NCTI: Noncompressible torso injury
PPE: Personal protective equipment
KAP: Knowledge attitude and practice
AT: Abdominal Tourniquet
MABC: Modified Adjustable Bar Clamp
CRoC: Combat Ready Clamp
AAJT: Abdominal Aortic and Junctional Tourniquet
REBOA: Resuscitative Endovascular Balloon Occlusion of the Aorta
JETT: Junctional emergency treatment tool
SJT: SAM junctional tourniquet
AJD: Adonis Junctional Device
CTH: Compressible torso hemorrhage
NCTH’: Remaining noncompressible torso hemorrhage
SBP: Systolic blood pressure
